# Dietary interventions and glycaemic control in type 2 diabetes: A systematic review and meta-analysis

**DOI:** 10.4102/jphia.v16i1.1325

**Published:** 2025-06-26

**Authors:** Anesu Marume, Exgratia Chidoko, Joconiah Chirenda

**Affiliations:** 1 Department of Global Public Health and Family Medicine, Faculty of Medicine and Health Sciences, University of Zimbabwe, Harare, Zimbabwe

**Keywords:** dietary patterns, diabetes, dietary interventions, T2DM, glaecemic control, nutritional strategies, non-communicable diseases

## Abstract

**Background:**

Advances in science and technology have significantly improved global living conditions, enhancing overall quality of life. However, these changes have also contributed to lifestyle shifts marked by reduced physical activity, increased sedentary behaviour, and altered dietary patterns fueling overnutrition and related non-communicable diseases (NCDs). Among these, type 2 diabetes mellitus (T2DM) has increased sharply over the past three decades, placing a burden on healthcare systems.

**Aim:**

This meta-analysis investigates the effectiveness of dietary interventions in managing T2DM and identifies nutritional strategies associated with improved glycaemic outcomes.

**Setting:**

The review includes studies conducted globally in community and health facility settings.

**Method:**

Peer-reviewed articles published between January 2010 and December 2024 were retrieved from PubMed, Scopus, and Web of Science. Eligible studies focused on dietary interventions for T2DM management. A random-effects model was used for meta-analysis, with effect sizes computed using R Studio. Heterogeneity was assessed using the *I*^2^ statistic.

**Results:**

Eighteen studies met the inclusion criteria. Dietary interventions significantly improved glycaemic control (mean difference: −0.30%; 95% CI: –0.45 to –0.15), despite high heterogeneity (*I*^2^ = 93.4%). Interventions focused solely on diet showed a modest effect (MD: –0.17%; 95% CI: –0.33 to –0.00), while intensive lifestyle interventions demonstrated a significantly higher impact (MD: –0.25%; 95% CI: –0.41 to –0.09).

**Conclusion:**

This study reinforces the critical role of lifestyle modifications particularly dietary changes and increased physical activity in managing T2DM. Comprehensive lifestyle interventions are more likely to yield meaningful improvements in glycaemic control.

**Contribution:**

This study highlights the importance of developing and scaling up multifaceted, sustainable strategies to support long-term lifestyle change in individuals living with T2DM.

## Introduction

Diet-related non-communicable diseases (NCDs), such as cardiovascular diseases, diabetes, and some cancers, are now considered major contributors to global morbidity and mortality. The World Health Organization (WHO) reports that diet-related NCDs are responsible for more than 70% of deaths worldwide each year.^1^ Among these, type 2 diabetes mellitus (T2DM) has seen an alarming rise, with its global prevalence quadrupling over the past three decades, disproportionately impacting low- and middle-income countries (LMICs).^2^ An estimated 828 million adults worldwide were reported to have T2DM in 2022, with 104 million estimated to be living in high-income countries while the majority resides in LMICs.^2^

Research has attributed the rising incidence and prevalence of T2DM to genetic factors, demographic shifts, socio-cultural changes, and evolving lifestyle patterns.^3,4,5^ The socio-cultural shift, economic growth, and globalisation have altered the landscape of risk and health behaviours.^6,7,8^ In many settings, these shifts have contributed to increased exposure to environmental and behavioural risk factors such as smoking, physical inactivity, and stress, compounding the metabolic disruptions that predispose individuals to T2DM.^9^

Poor dietary habits, particularly the excessive intake of energy-dense, nutrient-poor foods, play a central role in the aetiology of T2DM.^10^ Diets high in refined carbohydrates, added sugars, and unhealthy fats are closely associated with weight gain, insulin resistance, and chronic inflammation, all of which contribute to the development of T2DM.^11.12,13^ Conversely, diets rich in whole grains, lean proteins, unsaturated fats, and fibre have been shown to reduce the risk of T2DM and improve glycaemic control in those already diagnosed.^10,12^ In addition, inadequate key micronutrients such as magnesium, vitamin D, chromium, zinc, and vitamin B12 may impair glucose regulation and hinder effective diabetes management.^14,15,16,17^ Suboptimal levels of macro- and micro-nutrients have been associated with increased diabetes risk.

The nutrition transition, a phenomenon observed globally but particularly prominent in LMICs, provides critical insight into the dietary risk factors for T2DM.^18,19^ Nutrition transition refers to shifting from the traditionally consumed dietary regimen to a global, often Western-style one.^7^ In most countries, the traditional pattern is usually a nutrient-dense diet, while processed foods, high sugar content, and unhealthy fats characterise the emerging pattern.^20,21^ Rapid urbanisation, economic growth, and globalisation have accelerated this dietary transformation, particularly in regions where traditional diets were once protective against metabolic diseases.^20^ The resulting imbalance between caloric intake and expenditure has fuelled the obesity epidemic, a significant precursor to T2DM.^21^

Management of type 2 diabetes mellitus (T2DM) typically requires a lifelong commitment and involves a multifaceted approach, including lifestyle modifications, medication, and regular blood glucose monitoring. While medication, such as metformin, plays a critical role in stabilising blood glucose levels, sustainable lifestyle changes are the cornerstone of effective T2DM management. Dietary adjustments and regular physical activity support blood glucose control and avoid the potential complications and side effects often associated with prolonged medication use. This underscores the importance of exploring non-medical strategies that offer holistic benefits and address the root causes of diabetes rather than solely focusing on symptom management.

Research indicates that nutrition therapy for T2DM effectively enhances glycaemic control.^22^ The primary focus of dietary interventions has been to reduce energy intake by reshaping diets towards nutrient-rich foods such as fruits, vegetables, and whole grains, while minimising processed foods, sugary drinks, and saturated fats. Furthermore, individuals are encouraged to practise portion control, consume small and frequent meals, and avoid leading sedentary lifestyles. These measures enhance insulin sensitivity and help regulate blood sugar levels, improving long-term T2DM management.

Various dietary patterns have been proposed for managing T2DM, the most notable being the Mediterranean diet, the Dietary Approaches to Stop Hypertension (DASH) diet, very low-calorie diets, and diets low in saturated fat. Numerous trials have been conducted to assess the effectiveness of these approaches in controlling T2DM. This systematic review and meta-analysis aims to synthesise evidence from these studies to determine the role of diet in managing T2DM. By emphasising its potential to improve health outcomes, this research highlights the critical need for cost-effective and sustainable strategies to alleviate the financial burden of T2DM prescriptions and mitigate the risk of catastrophic health expenditures for individuals and healthcare systems globally.

## Methods

This systematic review adhered to the Preferred Reporting Items for Systematic Reviews and Meta-Analyses (PRISMA) guidelines.^23^

### Literature sources and search strategy

Relevant literature was identified through comprehensive searches of PubMed, Embase, CINAHL, and Web of Science databases. The search was conducted for studies published between January 2010 and December 2024. The search strategy included the terms: ‘diabetes mellitus type 2’, ‘obesity’, ‘randomised controlled trial’, ‘diet’, and ‘dietary patterns’. Boolean operators (AND, OR) were applied to refine the search. medical subject headings (MeSH) terms, keywords, and synonyms were used to ensure a wide coverage of relevant studies. Reference lists of identified studies and relevant reviews were examined for potential additional studies.

### Inclusion and exclusion criteria

Inclusion criteria for this review encompassed studies focusing on adult human participants diagnosed with T2DM. Eligible studies examined dietary interventions or patterns and reported outcomes related to glycaemic control (HbA1c and fasting glucose), weight management (body weight, height, body mass index [BMI]), or other health markers commonly associated with T2DM, such as blood pressure, lipid profile, insulin sensitivity, and inflammatory markers. Randomised controlled trials (RCTs), implementation science research, and quasi-experimental studies were eligible for inclusion. Studies that did not report on glycaemic control were excluded. Furthermore, studies were excluded if they were not published in English, were non-peer-reviewed articles, conference abstracts, grey literature, or involved paediatric populations or non-human subjects. In instances where one trial had multiple articles, the article reporting results satisfying the most conditions of the inclusion criteria was considered.

### Study selection process

**FIGURE 1:**
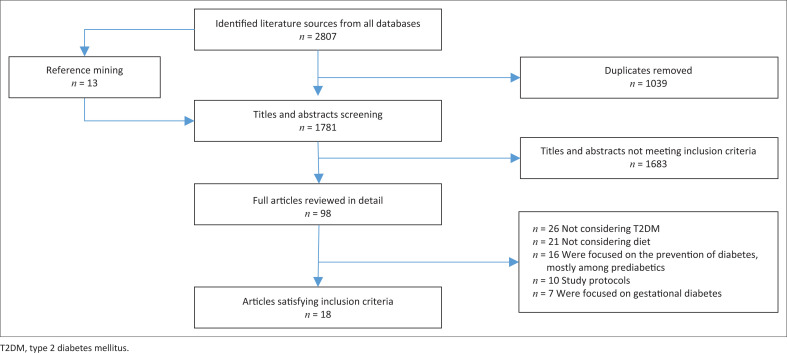
Flow diagram of study selection.

Two independent reviewers at postgraduate level conducted the selection process in three stages. Firstly, title screening was performed to remove irrelevant or duplicate records. Secondly, abstract screening was conducted to apply the inclusion and exclusion criteria and shortlist studies. Finally, full-text screening ensured the remaining studies met all predefined eligibility criteria. Any discrepancies were resolved through discussion, with the principal investigator mediating unresolved disagreements. A Preferred Reporting Items for Systematic Reviews and Meta-Analyses (PRISMA) flow diagram illustrating the selection process is presented in Figure 1.

### Data extraction

A standardised data-charting form (Online Appendix 1), collaboratively developed by the reviewers, was used to ensure consistency in data collection. The following details were extracted: study title and first author, publication year and country, study design, sample size, participant characteristics (age, gender), intervention and control conditions, outcome measures, baseline and follow-up HbA1c outcomes and key findings. When data was unclear or incomplete, the reviewers attempted to contact the corresponding authors. The study was excluded if no response was received or no clarifying information was available.

### Quality assessment

The Cochrane Risk of Bias tool (RoB 2) assessed the methodological quality of the included randomised controlled trials.^24^ This tool evaluates the following domains: bias arising from the randomisation process, bias due to deviations from intended interventions, bias due to missing outcome data, bias in the measurement of outcomes, and bias in the selection of the reported result. Each study was classified as ‘low risk’, ‘moderate risk’, or ‘high risk’ for each domain. The principal investigator resolved disagreements between reviewers through discussion or adjudication.

### Data synthesis and analysis

The extracted data was synthesised narratively to summarise the key findings from the included studies. HbA1c values at 6 months and 12 months were extracted for analytical purposes. Intervention types were divided into three categories: diet only, minimal lifestyle, and intensive lifestyle intervention. The authors developed the categories based on a thematic analysis of intervention characteristics reported across studies. The ‘diet only’ category includes studies that examined dietary packages, with or without accompanying health education. The ‘minimal lifestyle intervention’ category refers to studies that included a dietary component, low-intensity exercises, and health education. Lastly, the ‘intensive lifestyle interventions’ category includes studies incorporating a dietary component, high-intensity exercises, and behavioural interventions, such as goal setting (Online Appendix 1).

Quantitative synthesis was conducted using the meta package in R Studio.^25^ A random-effects model was applied for the meta-analysis to account for possible variability across studies. Heterogeneity among studies was evaluated using the *I*^2^ statistic, with values exceeding 50% indicating significant heterogeneity. The statistical significance of heterogeneity was evaluated using *p*-values, and sources of variability were explored and subdivided by follow-up period. The meta-analyses’ results are presented in forest plots, showing individual study mean differences (MDs) and 95% confidence intervals (CIs) alongside the pooled estimates. The weight of each study in the pooled analysis was determined by its sample size and variance.

### Ethical considerations

Ethical clearance to conduct this study was obtained from the Medical Research Council of Zimbabwe (No. MRCZ/A/3181).

## Results

The initial search uncovered 2807 studies, and after removing 1039 duplicates, 1781 studies remained. Screening the titles and abstracts eliminated 1683 studies that did not meet the inclusion criteria. A detailed review of 98 full-text articles excluded 72 studies for various reasons: 20 did not focus on diet, 23 were not specific to T2DM, 9 were study protocols, 6 concentrated on T2DM prevention, and 7 addressed gestational T2DM. In the end, 18 RCTs satisfied the inclusion criteria and were reviewed (Figure 1).

### Description of studies

The majority of studies were conducted in the United States (US) (*n* = 5), followed by Spain (*n* = 3) and Austria (*n* = 2), with single studies in China, Brazil, Denmark, England, Japan, Korea, Taiwan, and Canada. Follow-up periods varied, with 12 months being the most common (6 studies), while others ranged from as short as 4 weeks to as long as 46 months. Sample sizes varied widely, from as few as 12 participants to as many as 327. The mean age of participants across studies spanned from 20 years to 85 years, reflecting diverse adult populations with type 2 diabetes mellitus (T2DM). Attrition rates varied substantially, ranging from as low as 3% to as high as 40%, with some studies reporting slightly higher attrition in the control groups. This variation often reflected differences in study duration, population characteristics, and intervention demands (Table 1).

**TABLE 1:** Characteristics of studies considered in the review.

Study	Country	Period (months)	Intervention				Control				Inclusion criteria
			*n*	Attrition	Age Mean	s.d.	Number	Attrition	Age Mean	s.d.	
Alonso-Dominguez^26^	Spain	12	102	8	60.8	7.8	102	11	60.4	8.4	Fasting plasma glucose > 126 mg/dL, HbA1c > 6.5%, no known condition that interferes with adherence to interventions.
Celli^27^	US	12	50	9	71.4	3.7	50	7	72.3	4.0	Age 65–85 years, verified diagnosis of T2DM, BMI ≥ 27 kg/m^2^, sedentary lifestyle, stable body weight (± 2 kg), stable medication use < 6 months, HbA1c < 11%, no known condition that interferes with adherence to intervention.
Goday^28^	Spain	4	45	5	54.89	8.81	44	8	54.17	7.97	Age 30–65 years, BMI 30–35 kg/m^2^, HbA1c ≥ 9%, T2DM diagnosis < 10 years, not on insulin therapy, no impaired renal or liver function, no known condition that interferes with adherence to interventions.
Goode^29^	Australia	24	135	33	57.1	7.3	144	20	56.8	9.3	Age 20–75 years, BMI ≥ 25 kg/m^2^, Telephone, not on weight-loss medications or previous/planned bariatric surgery, no known condition contraindication to unsupervised physical activity.
Ha^30^	Canada	6	143	8	62.1	0.7	124	23	61.8	0.9	HbA1c 6.5% – 8.0% (DMGI trial) and 6.5% – 8.5% (DM-MRI trial), a stable dose of oral antihyperglycaemic agents for at least 2 months (DM-MRI) or 3 months (DMGI).
Johansen^31^	Denmark	12	64	2	53.6	9.1	34	3	56.6	8.1	T2DM diagnosis < 10 years, BMI 25–40 kg/m^2^, ≤ 2 glucose-lowering medications, HbA1c ≤ 9%, non-IDD, no complications such as diabetic retinopathy or nephropathy.
Kitazawa^32^	Japan	3	86	7	49.19	8.75	82	5	47.38	7.49	HbA1c 5.6% – 6.4%, FBG 110–125 mg/dL, BMI 23–40 kg/m^2^, smartphone and able to use the Health2Sync app, no known condition that interferes with adherence to interventions, taking corticosteroids.
Lee^33^	Republic of Korea	3	46	7	57.5	7.7	47	6	58.3	7.0	Use of HM ≥ 6 months, HbA1c 6.0% – 11.0%, no reported increase in dosage of HM or additional medication < 2 months, not a vegetarian, no known condition that interferes with adherence to interventions.
Lin^34^	Taiwan	6	58	2	NR	-	56	4	NR	-	Age 20–75 years, T2DM diagnosis ≥ 1 year, HbA1c ≥ 7.0%, no known condition that interferes with adherence to intervention.
Lynch^35^	US	18	106	7	55.1	11.5	105	8	54.8	9.0	BMI ≥ 18.5 kg/m^2^, HbA1c ≥ 7.0%, African-American, at least one visit to a participating primary care clinic in the past year, availability for group sessions, GFR ≥ 15 mL/min/1.73 m^2^, no history of weight-loss surgery, no known use of prednisone within 3 months, telephone access, no known condition that interferes with adherence to interventions.
Paula^36^	Brazil	1	20	NR	61.8	8.1	20	NR	62.5	8.8	Office BP ≥ 140/90 mmHg, daytime ABPM ≥ 135/85 mmHg, BMI ≤ 40 kg/m^2^, Serum creatinine < 176 mmol/L, not working night shift, no secondary causes of hypertension, BP ≤ 180/120 mmHg, no known condition that interferes with adherence to interventions.
Salas-Salvado^37^	Spain	12	327	9	66	5	299	7	65	5.0	Men 55–75 years, women 60–75 years, BMI 27 kg/m^2^ – 40 kg/m^2^, no known CVD, at least 3 of abdominal obesity, elevated triglycerides, reduced HDL cholesterol, elevated blood pressure, and elevated fasting glucose.
Sampson^38^	England	46	141	24	64.1	9.9	149	8	63.5	10.0	Age ≥ 40 years, BMI ≥ 30 kg/m^2^ or Age ≥ 40 years, BMI ≥ 25 kg/m^2^ and family history of T2DM, FPG ≥ 7.0 mmol/L, HbA1c of ≥ 48 mmol/mol, single OGTT = extreme hyperglycaemia.
Saslow^39^	US	8	12	1	53.0	10.2	13	1	58.2	6.7	BMI ≥ 25, HbA1c 6.5% – 9.0%; Regular Internet access, not on any T2DM medication other than metformin, no known condition that interferes with adherence to interventions.
Saslow^40^	US	4	23	1	60.09	6.03	25	3	58.40	8.11	Age 21–70 years, HbA1c ≥ 5.7% within the previous 12 months, BMI 25–50 kg/m^2^, SBP ≥ 130 mm Hg, not using any of insulin, phenytoin, lithium, steroids, immunosuppressants, or warfarin, not part of any formal weight-loss programme or use of weight-loss drugs, not a vegan or vegetarian, no known condition that interferes with adherence to interventions.
Taheri^41^	Qatar	12	70	-	41.9	5.4	77	-	42.3	5.8	Age 18–50 years, verified T2DM < 3 years, BMI ≥ 27.0 kg/m^2^, resident of MENA, no known condition that interferes with adherence to intervention.
Tay^42^	Australia	12	58	17	58.0	7.0	57	21	58.0	7.0	BMI 26–45, HbA1c ≥ 7.0% or use of T2DM medication, no known condition that interferes with adherence to interventions.
Turner-McGrievy^43^	US	3	22	3	49.4	11.3	21	4	46.8	11.2	Age 18–65 years, BMI 25.1–49.9 kg/m^2^, ability to attend online classes and complete assessments, not following diet being examined, not enrolled in weight-loss programme, no known condition that interferes with adherence to interventions.
Wang^44^	China	3	24	2	66.79	9.12	25	3	61.20	11.71	HbA1c > 6.0%, T2DM diagnosis < 2 years, BMI 27–50 kg/m^2^, willingness to lose weight, no known condition that interferes with adherence to interventions.

Note: Please see the full reference list of the article, Marume A, Chidoko E, Chirenda J. Dietary interventions and glycaemic control in type 2 diabetes: A systematic review and meta-analysis. J Public Health Africa. 2025;16(1), a1325. https://doi.org/10.4102/jphia.v16i1.1325, for more information.

FBG, fasting blood glucose; FPG, fasting plasma glucose; HM, hypoglycaemic medication; IDD, insulin dependent; OGTT, oral glucose tolerance test; type II diabetes mellitus; HbA1c, glycated haemoglobin; T2DM, type 2 diabetes mellitus; US, United States; DMGI, diabetes management with a low glycemic index diet; DM-MRI, diabetes management - magnetic resonance imaging; GFR, glomerular filtration rate; ABPM, ambulatory blood pressure monitoring; FPG, fasting plasma glucose; SBP, systolic blood pressure.

### Quality of studies

All studies included in this review reported conducting randomisation before assigning participants to either the control or experimental group. The majority found that it was challenging to blind the participants, as the participants would inherently know if they were in the experimental group once they are described the instructions to follow. However, some studies addressed this limitation by blinding participants regarding whether the trial included an experimental or control group. In contrast, others kept them unaware of the specific conditions of each group.

Most studies reported blinding the statistician, although a few did not specify whether a statistician was involved in the randomisation process. A number of studies did not report key outcomes, such as BMI, HbA1c, weight changes, or participant age, revealing gaps in reporting completeness. Notably, none of the reviewed articles provided evidence of selective reporting.

### Dietary intervention

Most of the studies were two-arm designs, comparing the effect of one dietary intervention to a different form of dietary intervention or one intervention against usual care, with or without minimal additional services. Three studies included more than two intervention arms: one evaluated three distinct dietary interventions,^43^ another compared two types of interventions versus a single control,^38^ and the third examined the relationship between two dietary patterns, both with and without additional support activities.^40^ Some studies did not consider specific dietary patterns but either considered individualised dietary recommendations or specific nutrition education that sought to reduce the consumption of certain foods, more often high-calorie dietary options. The most common additional support for dietary interventions was physical exercise, health coaching, goal setting, self-monitoring with or without exercise, diet, weight, or glucose technology. Less common inclusion in the interventions was social support, either through peer support or community support Online Appendix 1.

Most dietary patterns aim to reduce the consumption of carbohydrates more often to achieve nutrition ketosis.^28,39,40,42,44^ This was mainly achieved by identifying food items with low carbohydrates or replacing carbohydrates from the diet. Among the studies that targeted nutritional ketosis, some monitored this by providing participants with ketone test kits to measure the extent to which they were able to adhere to the recommendations.^40^ Among the established dietary patterns, the Mediterranean Dietary Pattern,^28,30,32^ was the most commonly considered, followed by the DASH dietary pattern,^36,40^ and the Vegan Dietary pattern^33,43^ (Table 2).

**TABLE 2:** Dietary interventions characteristics.

Study	Med	DASH	H-US	Veg	Low carb	VLCK	Low fat	LGI	High fibre	Nutrition education	Create your plate
Alonso-Dominguez^26^	X	-	-	-	-	-	-	-	-	O	-
Celli^27^	-	-	-	-	X	-	-	-	-	O	-
Goday^28^	-	-	-	-	O	X	-	-	-	-	-
Goode^29^	-	-	-	-	X	-	-	-	-	O	-
Ha^30^	-	-	-	-	-	-	-	X	O	-	-
Johansen^31^	-	-	-	-	-	-	-	-	-	O	X
Kitazawa^32^	-	-	-	-	-	-	-	-	-	XO	-
Lee^33^	-	-	-	X	-	-	-	-	-	O	-
Lin^34^	-	-	-	-	-	-	-	-	-	O	-
Lynch^35^	-	-	-	-	X	-	-	-	-	O	-
Paula^36^	-	X		-	-	-	-	-	-	O	-
Salas-Salvado^37^	X	-	-	-	X	-	-	-	-	-	-
Sampson^38^	-	-	-	-	-	-	X	-	-	O	-
Saslow^39^	-	-	-	-	-	X	O	-	-	-	O
Saslow^40^	-	O	-	-	-	X	-	-	-	-	-
Tay^42^	-	-	-	-	-	X	-	O	-	-	-
Turner-McGrievy^43^	X	-	-	O	-	-	-	-	-	-	-
Wang^44^	-	-	-	-	-	X	O	-	-	-	-

Note: Please see the full reference list of the article, Marume A, Chidoko E, Chirenda J. Dietary interventions and glycaemic control in type 2 diabetes: A systematic review and meta-analysis. J Public Health Africa. 2025;16(1), a1325. https://doi.org/10.4102/jphia.v16i1.1325, for more information.

X, primary intervention; O, intervention served as the control or comparator; □, third arm intervention.

Create your plate, a diabetes-focused meal-planning intervention; DASH, dietary approaches to stop hypertension; H-US, Healthy United States - style eating pattern; LGI, low glycaemic index diet; Low carb, low-carbohydrate diet; low fat, low fat diet; Med, Mediterranean diet; Nutrition education, dietary counselling or education provided; Veg, vegetarian diet; VLCK, very low-carbohydrate ketogenic diet.

### Additional components to dietary interventions

**FIGURE 2:**
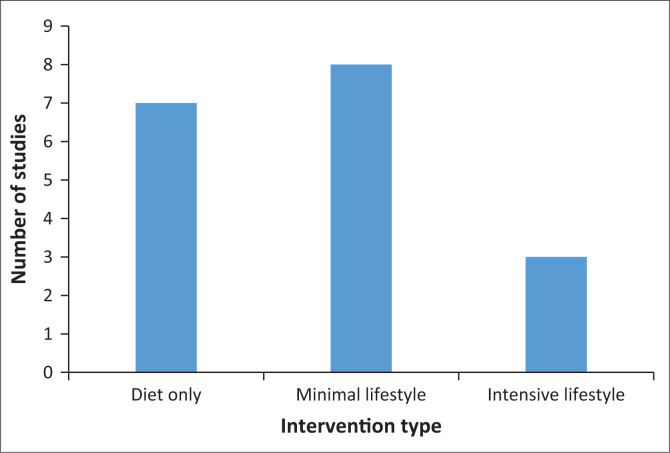
Intervention category.

Seven studies were classified as focusing on diet only, while the majority included additional lifestyle components with varying intensities (Figure 2). Two studies concentrated exclusively on diet and its impact on T2DM,^30,40^ but most combined dietary interventions with behavioural support, nutritional education, or physical exercise. Among the studies reporting exercise, the type of activity varied, although specifics were often lacking. Aerobic and resistance exercises were mentioned in some cases while walking (e.g., 10 000 steps daily, monitored with pedometers) emerged as the most common physical activity.^26,31,36^ Four studies did not report any exercise components.^4,28,30,43^

Behavioural support activities were an important additional component, although not all trials provided detailed descriptions of the interventions. General nutrition education, medical checkups, and group or individual counselling were frequently included. Some studies implemented specific strategies like health coaching, mobile phone counselling sessions, or goal setting to improve adherence to dietary regimens and exercise plans.

For the control groups, most studies incorporated a health education component alongside the usual care provided at health facilities. Usual care often included health and nutrition education, ranging from brief interventions, such as a single orientation day,^26,35,38^ to more prolonged engagements.^27,29,31^ In some cases, usual care involved adherence to dietary recommendations from national associations, such as the American Diabetes Association.^33,36,40^ However, a few studies did not provide sufficient details on the usual care provided to control groups.^32,34^

### Impact of interventions on glycated haemoglobin level

**FIGURE 3:**
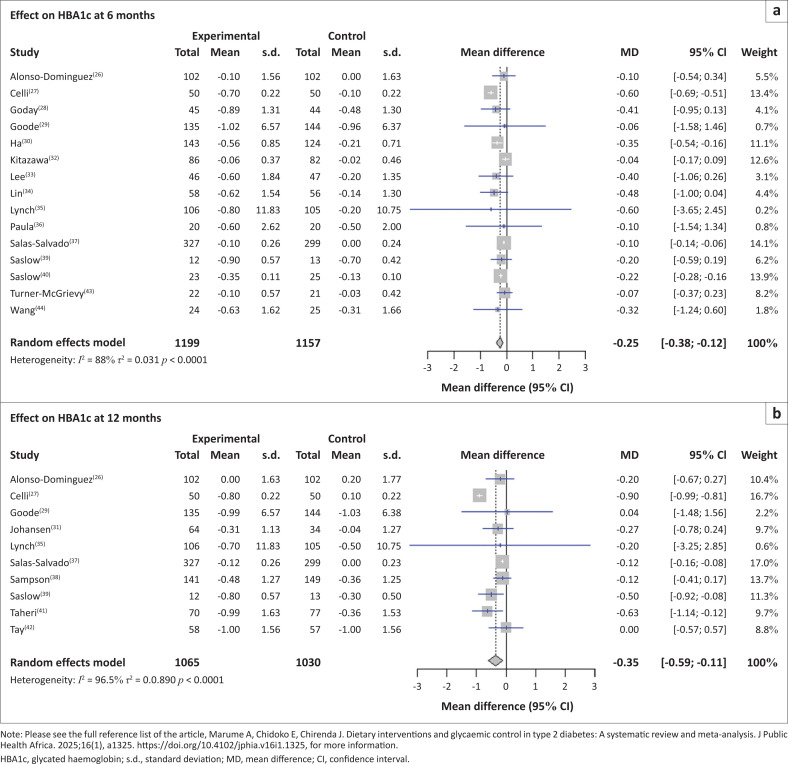
Forest plot analysis of the effect of interventions on HbA1c outcomes by time.

A total of fourteen studies reported HbA1c outcomes at 6 months. The 14 studies reported a pooled MD of −0.25% (95% CI: –0.38 to –0.12, *p* < 0.0001) with high heterogeneity (*I*^2^ = 88.0%). Ten studies reported HbA1c outcomes at 12 months. Among the studies that reported 12 months of intervention reported a pooled MD of –0.35% (95% CI: –0.59 to –0.11, *p* < 0.0001), with high heterogeneity (*I*^2^ = 96.5%) (Figure 3).

**FIGURE 4:**
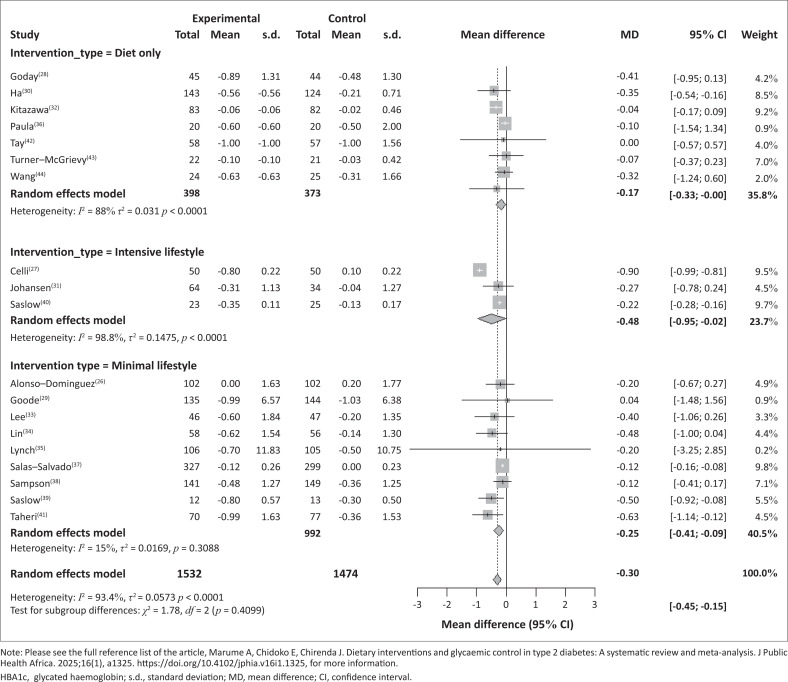
Intervention subgroup analysis for the effect of interventions on HbA1c.

In subgroup analyses by intervention type for the studies whose intervention was largely diet only, the pooled MD was –0.17 (95% CI –0.33, –0.00), suggesting a mild but statistically significant improvement. The heterogeneity within this subgroup was moderate (*I*^2^ = 31.8%, *p* = 0.1849), indicating a relatively consistent effect across the studies. Among the three studies assessing minimal lifestyle interventions the pooled MD was –0.48 (95% CI: –0.95, –0.02), reflecting a moderate and statistically significant effect. However, heterogeneity was high (*I*^2^ = 98.8%, *p* < 0.0001), indicating substantial variation among the study results. The last group of studies, intensive lifestyle interventions, yielded reported a pooled MD of –0.25 (95% CI: –0.41, –0.09), indicating a small but significant effect with low heterogeneity (*I*^2^ = 15%, *p* = 0.3088), suggesting that the studies produced relatively consistent results (Figure 4).

Overall, the pooled analysis showed a statistically significant MD of –0.30 (95% CI: –0.45, –0.15) with high heterogeneity noted (*I*^2^ = 93.4%, *p* = 0.4099), reflecting study variability. The test for subgroup differences (χ^2^ = 1.78, *df* = 2, *p* = 0.4099) did not reveal statistically significant differences among the three intervention types (Figure 4). Therefore, while all interventions appeared to provide some benefit, no single approach demonstrated a significantly superior outcome to the others.

## Discussion

This systematic review identified 18 studies that considered the effect of diet on diabetes. The meta-analysis provides evidence on the impact of various dietary interventions on glycaemic control. Pooled analyses by time and intervention model show that diet positively reduces HbA1c, with varying levels by both subgroup and time factor. The study argues that while there is evidence of diminishing returns over time, and by the overall intervention mode, interpretation of the results must account for the considerable variability among the included studies, as evidenced by the high heterogeneity indices.

The distinction in outcomes between short-term and long-term follow-up studies is a notable finding. Mechanistically, short-term improvements may be attributed to immediate changes in insulin sensitivity, weight loss, or reductions in postprandial glucose excursions often observed shortly after dietary modifications. Adherence to dietary interventions is likely to decline over extended periods, diminishing their effectiveness. In addition, metabolic adaptations may counteract the initial benefits, such as compensatory appetite increases or energy expenditure changes, which may explain the observed differences in HbA1c outcomes between short-term and long-term studies.^45,46,47,48^ However, other RCTs argue that dietary changes do not result in compensatory appetite increase but may result in motivation not to eat.^49^ Adherence to dietary interventions is a well-documented challenge in clinical trials.^50,51^ Over time, participants may revert to their habitual eating patterns, mainly if the interventions are restrictive or lack ongoing support.^9^ This decline in adherence could partially explain the diminishing effect observed in long-term studies.

There were varied differences in the design of interventions observed, which can influence participant’s adherence, access to dietary options, and the sustainability of dietary changes over time. For participants to follow restrictive dietary patterns, such as ketogenic, vegan, or DASH diets, often demand significant changes to habitual eating behaviours. These changes may be compounded by practical barriers such as limited access to the required foods or the higher cost of adhering to specific diets. For instance, ketogenic diets, which often emphasise high-fat and low-carbohydrate foods, may involve specialty items that are less accessible or affordable for some populations. Similarly, adherence to the Mediterranean diet is influenced by the availability of fresh fruits, vegetables, and olive oil, which can vary by region and socio-economic status. These access and cost barriers underscore the need to tailor dietary recommendations to the socio-economic and cultural contexts of the target population. Such challenges in adherence and accessibility are critical when evaluating the long-term sustainability of dietary interventions.

Most studies report incorporating behavioural support mechanisms, such as health coaching, self-monitoring, and peer support, to enhance adherence to dietary interventions. These mechanisms are often complemented by physical exercise, an additional component to strengthen the intervention’s overall impact. This study provides evidence on the impact of additional support with intensive lifestyle changes having the most substantial relationship with glycaemic control. This is supported by earlier reviews, which suggest that interventions targeting a single attribute tend to have a limited effect on glycaemic control compared to those integrating multiple components.^52^ For instance, interventions combining dietary modifications with exercise, behavioural counselling, and self-monitoring have significantly improved glycaemic outcomes. This multidimensional approach, often called intensive lifestyle interventions (ILIs), achieves greater success because of its synergistic effects. By addressing several aspects of lifestyle simultaneously, these interventions improve initial outcomes and support the maintenance of healthy behaviours over time. Including multiple components helps participants overcome common barriers, such as the lack of motivation or the challenges of sustaining dietary changes in isolation. Furthermore, the interaction between these components, such as the role of physical activity in enhancing insulin sensitivity alongside dietary adjustments, contributes to more comprehensive improvements in metabolic health.

Behavioural support, in particular, has been identified as a critical factor in sustaining adherence, as it provides ongoing reinforcement, goal setting, and problem-solving strategies tailored to individual needs.^22,52^ In contrast, interventions focusing solely on one or a few aspects may lack the holistic approach necessary to address the complex interplay of factors influencing glycaemic control, as seen with the minimal lifestyle interventions in this review. These findings underscore the importance of designing multifaceted interventions that account for the immediate and long-term needs of individuals managing T2DM. However, the type and intensity of these components vary widely, ranging from structured programmes with frequent interactions to less intensive approaches with limited participant contact. Studies that combine dietary interventions with physical activity also differ in the specificity and monitoring of prescribed exercises. For example, some studies include detailed exercise regimens, while others recommend increased physical activity without clear guidance. These inconsistencies make it challenging to isolate the effects of dietary interventions and compare findings across studies.^22,53^

Although the average reduction in HbA1c observed across studies was a modest 0.3%, it remains clinically meaningful, particularly given that it was achieved through lifestyle interventions rather than pharmacotherapy. Recent meta-analyses and trials have reported comparable reductions in HbA1c ranging from 0.3% to 0.5% in response to diet and physical activity interventions, underscoring the consistency of these findings.^54,55,56^ Importantly, even modest improvements in glycaemic control are associated with a lower risk of microvascular complications over time, reinforcing the clinical relevance of such lifestyle-driven changes.^57^

### Strengths and limitations

The study used a systematic and rigorous narrative synthesis approach, which allowed for a detailed examination of intervention characteristics beyond what is typically captured in meta-analysis alone. The quantity of articles identified was limited, and significant discrepancies existed within the intervention-control group relationship. Consequently, this challenges the assessment of consistency between direct and indirect comparisons. Similarly, while we managed to conduct subgroup analyses based on intervention type, we did not go further to do subgroup analyses based on population characteristics, follow-up period, and study quality, which may affect the generalisation and interpretation of our findings. These analyses could have provided deeper insights into factors influencing dietary intervention effectiveness. In addition, the heterogeneity in study designs, differences in outcome measurement timeframes, and varied levels of adherence limited the comparability of results. The lack of standardised reporting across studies further complicates efforts to aggregate data meaningfully.

## Conclusion

The findings support the effectiveness of dietary interventions in achieving modest but clinically meaningful short-term reductions in HbA1c. However, the sustainability of these effects over time remains uncertain. Interventions that consider patient preferences, support adherence, and are adaptable to diverse socio-economic contexts are essential to maximise long-term benefits.

### Recommendations

Future studies could consider the effect of differences by intervention type. A majority of the studies had a maximum follow-up of 12 months, while to be able to conclude maintenance of behaviour, extended follow-up may be required, and then assess the persistence of intervention effects. Interventions should be designed flexibly to accommodate patient preferences, cultural context, and practical feasibility. Research should explore strategies such as digital support tools, behavioural counselling, and group-based interventions to enhance and sustain adherence. Further research is warranted to understand the mechanisms behind the waning of dietary intervention effects over time, including metabolic adaptation, behavioural fatigue, or environmental barriers. Nevertheless, future research should leverage the findings of this meta-analysis through long-term trials characterised by rigorous designs aimed at enhancing and evaluating the sustainability of dietary interventions over time. Such studies ought to incorporate strategies to foster adherence, including regular follow-ups, behavioural support, and the integration of digital health technologies. Moreover, research should investigate the mechanisms that contribute to the attenuation of effects over time.
